# Global Glaucoma Staging System (GGSS): A New Method to Simultaneously Assess the Severity of Both Functional and Structural Damage in Glaucoma

**DOI:** 10.3390/jcm10194414

**Published:** 2021-09-26

**Authors:** Paolo Brusini

**Affiliations:** Department of Ophthalmology Health Clinic “Città di Udine”, 33100 Udine, Italy; brusini@libero.it

**Keywords:** classification systems, chronic open-angle glaucoma (OAG), visual field, optical coherent tomography (OCT), structural and functional damage

## Abstract

Background: The classification of damage in glaucoma is usually based either on visual field or optical coherent tomography (OCT) assessment. No currently available method is able to simultaneously categorize functional and structural damage. Material and Methods: In this study, 283 patients with chronic open-angle glaucoma (OAG) at different stages and 67 healthy subjects were tested with both standard automated perimetry and spectral domain OCT for retinal nerve fiber layer (RNFL) assessment. The visual field data were classified using the Glaucoma Staging System 2, whereas OCT results were processed with the OCT Glaucoma Staging System. These data were used to create a new staging system (global glaucoma staging system, GGSS), in which the severity of visual field and RNFL damage is reported on the Y and X axis, respectively. The GGSS was tested in a different sample of 147 patients with manifest OAG, 56 with preperimetric glaucoma and 43 normal subjects. A six-stage clinical classification, based on the analysis of visual fields and optic disc appearance, was used as gold standard. Results: The GGSS was able to correctly classify in the same stage or within the immediately adjacent stages 145 cases on 147 (98.6%). Fifty-four preperimetric cases (96.4%) were classified as borderline or abnormal (Stage 1 or 2). Here, 41 normal eyes out of 43 were correctly classified as Stage 0, with a specificity of 95.3%. Conclusions: Preliminary results from this study are encouraging and suggest that the new GGSS is able to provide information concerning the severity of structural and functional damage in an integrated manner.

## 1. Introduction

Chronic open angle glaucoma (OAG) is a progressive disease which can slowly, but irreversibly, damage the patient’s optic nerve and visual function up to total visual impairment. Visual field and optic disc examination are thus important, both for an early diagnosis of glaucoma and for the definition of the severity of the disease. Visual field testing with standard automated perimetry (SAP) is currently the most commonly used method to quantify glaucomatous damage. On the other hand, modern spectral-domain optical coherence tomography (sd-OCT) instruments are able to accurately analyze the optic disc, the peripapillary retinal nerve fiber layer (RNFL), and the macular ganglion cells (RGCs), thus providing a better diagnostic ability for discriminating between healthy eyes and glaucomatous eyes.

Several staging methods for categorizing glaucomatous functional damage severity, and staging the RNFL damage assessed with the glaucoma diagnosis (GDx) or OCT, are currently available [1,2,3,4,5,6,7,8,9]. The most popular systems to stage the functional damage include: (1) the three-stage Hodapp–Parrish–Anderson method, which is based on the assessment of the mean deviation (MD) value, number of depressed points in the pattern deviation map, and proximity of defects to fixation point [3]; (2) the five-stage Mills et al. staging system, which is an evolution of the former [4]; (3) the AGIS method, which takes the number of depressed points in various areas in the central visual field into account, and provides a score ranging between 0 and 20 [5]; and, the glaucoma staging system 2 (GSS 2), based on the MD and pattern standard deviation (PSD) values plotted in an x-y graph [6]. A more detailed description of these methods can be found in our previous publication [1]. With regard to structural damage, three methods are currently available: (1) the GDx staging system, which divides the severity of nerve fiber loss into six stages, taking the values of the superior and inferior RNFL thickness into consideration [7]; (2) the three-stage method proposed by Elbendary and Elal, which is based on the average RNFL values assessed with OCT [8]; and (3) the six-stages OCT glaucoma staging system, which uses the superior and inferior quadrant RNFL thickness values [9]. All of these methods have limitations. With regards to functional damage classification, some methods are difficult to use and time consuming, while the GSS 2 uses only two parameters and does not consider the topographical location of the defect. With regards to structural damage classification, the GDx staging system is based on obsolete technology and does not take into consideration the patient’s age, such as the Elbendary and Elal method. The OCT glaucoma staging system is based on two parameters only and, similarly to the other methods, does not take functional damage into account. A further step toward a classification system, which takes these two types of damage into account at the same time, thus integrating the data arising from perimetry and from OCT, would be advantageous both in research and in daily clinical practice. 

The purpose of this study was to introduce a new and easy-to-use method, called the gobal glaucoma staging system (GGSS, patent pending), which plots structural and functional damage data on an x-y diagram in order to provide a standardized classification of the entire glaucomatous damage.

## 2. Materials and Methods

### 2.1. GGSS Design and Creation

This retrospective study included 283 patients (mean age 63.2 years ± 9.5) affected with either ocular hypertension (OHT; IOP > 21 mmHg; normal optic nerve head [ONH] and RNFL appearance, and normal visual field on SAP) or chronic OAG (IOP ≥21 mmHg before medication, abnormal appearance of the ONH or RNFL, and reproducible glaucomatous visual field defects); and 67 normal subjects (mean age 57.8 ± 9.3).

Inclusion criteria for glaucoma patients were: best corrected visual acuity higher than or equal to 0.7; absence of ocular pathology other than open-angle glaucoma and mild cataract; reliable SAP test results; and good OCT image quality.

Exclusion criteria included: ametropia > ±5 diopters, pupil diameter < 2 mm; secondary causes of glaucoma, apart from pseudoexfoliation and pigmentary glaucoma; normal tension-glaucoma cases; optic disc anomalies; large peripapillary atrophy; clear visual field or OCT artifacts; media opacities preventing good image scans; and neurological disorders.

Only one eye per subject was randomly selected if both eyes met the inclusion criteria. All subjects underwent a complete ophthalmologic examination, including SAP, and sd-OCT testing within a 3-month period. Normal subjects were recruited from staff members and volunteers to collect clinical data for other studies. OHT and OAG patients were recruited from the Glaucoma Center of the Department of Ophthalmology at Health Clinic “Città di Udine” of Udine, Italy. The study was in compliance with the tenets of the Declaration of Helsinki, with our Institutional Review Board (IRB) and HIPAA requirements of our institute. Informed consent prior to testing was obtained from normal subjects only, as tests previously performed on glaucomatous patients who were no longer reachable were used. No personal data, except for the age of patients, were directly linked to the data analysis or published results.

IOP was measured with Goldmann applanation tonometry (GAT). ONH was assessed by a glaucoma expert ophthalmologist with slit-lamp stereoscopic ophthalmoscopy using a 78-diopter lens. Glaucomatous optic neuropathy (GON) was defined in the presence of at least one of the following findings: ONH cupping; notching involving ≥2 clock hours; focal or diffuse atrophy of neural rim area involving ≥2 clock hours; disc hemorrhage not related to other disease; and focal or generalized atrophy of the RNFL. SAP was performed with the Zeiss–Humphrey field analyzer (HFA) II-i (Carl Zeiss Meditec, Dublin, CA, USA) using the 24-2 or 30-2 test with standard Swedish interactive thresholding algorithm (SITA) strategy. Reliability criteria for HFA tests included false-positive < 15%, false-negative < 33%, and fixation losses < 20%. The severity of visual field defects was classified using the glaucoma staging system 2 [6].

OCT was performed through an undilated pupil with three different OCT machines: (1) the Nidek RS-3000 Advance Capture sd-OCT, software version Navis-Ex 1.4.0.1 (Nidek, Gamagori, Japan); (2) the Zeiss Cirrus SW Ver.6.0.0.599 (Carl Zeiss Meditec, Dublin, CA, USA); and (3) the Topcon Triton swept-source OCT, software version 1.24 (Topcon Corp, Tokyo, Japan). The RNFL thickness measurements were performed using the Zeiss optic disc cube 200 × 200; the Nidek disc map protocol 6 × 6 mm; and the Topcon 3-D disc report. Only reliable results (Signal Strength Index ≥7/10 with no evident artifacts for Zeiss Cirrus and ≥6/10 for Nidek RS-3000; imaging quality value >30 for Topcon Triton) were considered in the analysis. Only one OCT was used during the creation phase of GGSS, which was based on the availability. When two (or three) different tests performed with different OCTs were available for the same patient, the test with the highest signal strength index or that with minor artifacts was used. In the cases of similar performances, the test was randomly chosen. The RNFL loss severity was classified using the OCT glaucoma staging system (OCT GSS) [9], which uses both the superior quadrant average and the inferior quadrant average values, taken from the RNFL quadrants (called TSNIT map in Nidek OCT) and plotted on the x-y axes diagram, respectively. Six curved lines divide the diagram into seven sectors: normal RNFL results are displayed in the superior-left corner of the graph, while RNFL defects are classified into six stages of increasing severity, from borderline to stage 5 (almost complete loss of RNFL).

**Figure 1:**
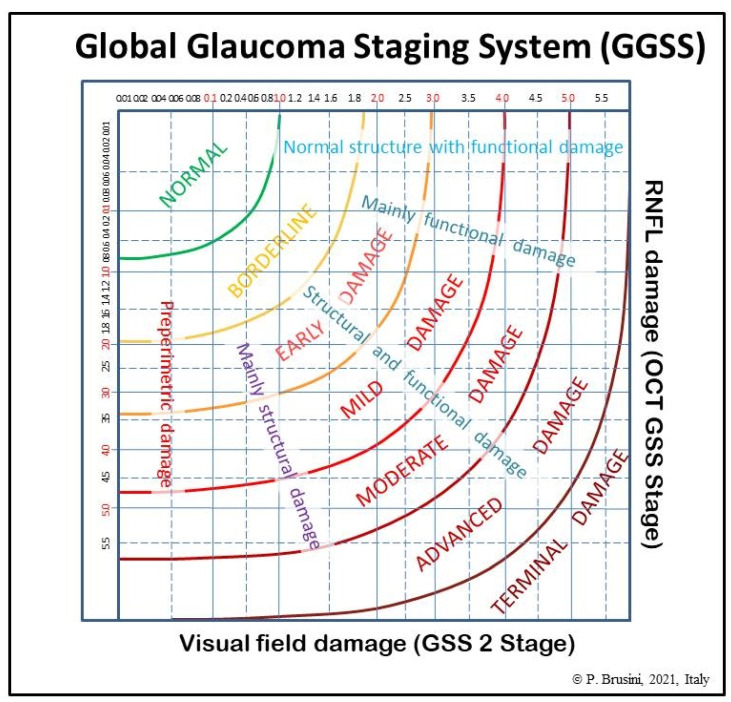
The global glaucoma staging system (GGSS). RNFL: retinal nerve fiber layer; OCT: optical coherent tomography; GSS 2: Glaucoma Staging System 2.

The global glaucoma staging system (Patent Pending; Patent Application No. 102019000006120) was designed for integrating data from both the GSS 2 (for functional damage) and the OCT GSS (for structural damage). A new graph was created in which visual field damage data lie on the x-axis and structural (RNFL) damage on the y-axis (Figure 1). 

The graph is divided into seven areas by curvilinear lines, based on the assessment of 350 clinical cases. Normal results are displayed in the superior-left corner. Glaucoma damage severity progressively increases toward the bottom right corner, including borderline results, early, mild, moderate, advanced, and terminal damage. For this purpose, continuous numbers (automatically calculated by the software) instead of the single stages were used, thus deleting the curvilinear lines which divide stages of different severity in the two previous systems. Moreover, five different sectors are present in the graph, which indicate: (1) preperimetric damage, in the left part, in which the visual field is completely normal; (2) mainly structural damage; (3) both structural and functional damage present in equal measure; (4) mainly functional damage; and (5) normal structure with functional damage, in the upper part. In this manner, a complete representation of the glaucomatous damage spectrum is provided.

**Figure 2:**
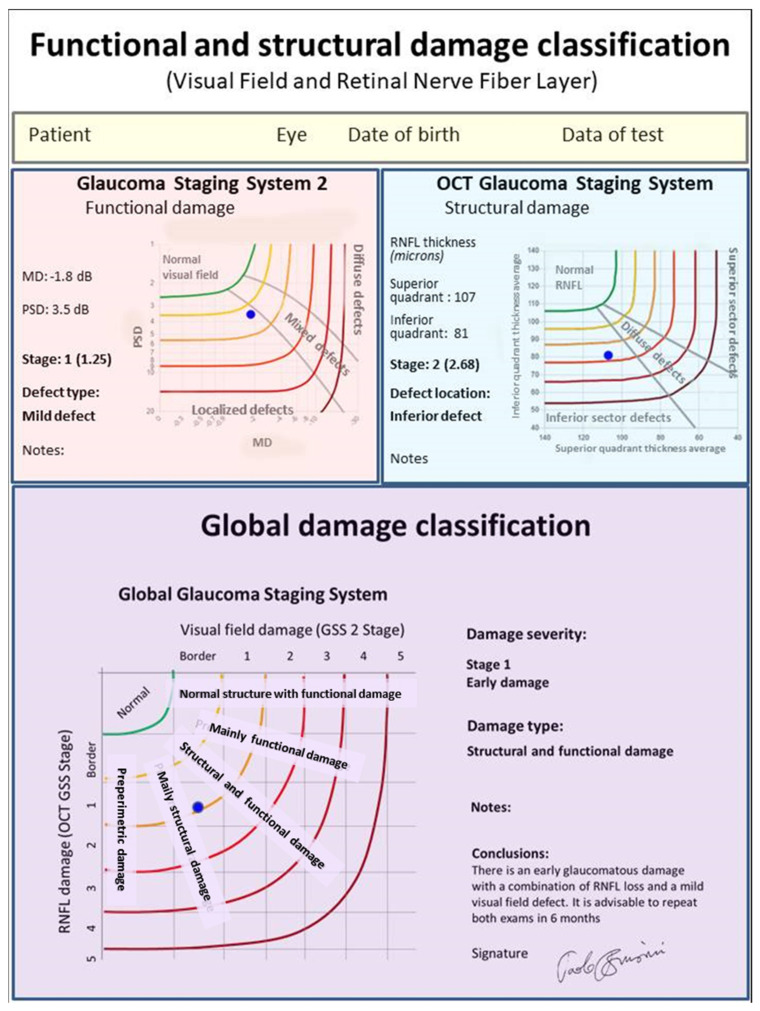
Printout obtained with the Glaucoma Staging System software. At the top of the page the results from both the GSS 2 and OCT GSS are displayed. At the bottom is the new GGSS display. MD: mean deviation; PSD: pattern standard deviation.

In order to obtain an easy-to-use classification method, a special software was created, which only requires one the entry of the following data: (1) the patient’s personal data, including date of birth and tested eye; (2) the visual field mean deviation (MD) and pattern standard deviation (PSD) (or squared loss variance (√LV) for Octopus perimeters) values; and (3) the superior and inferior quadrant RNFL values (in microns), as reported in the standard printout of almost any OCT instrument. Upon pressing the “calculate” button, both the stage of the global defect and its characteristics (prevalence or similarity of two types of damage) are immediately displayed, together with the GSS 2 and OCT GSS classifications. A comprehensive report can be printed and used either to provide a copy to the patient or to be entered in the patient medical records (Figure 2).

### 2.2. GGSS Validation

In order to assess the sensitivity and specificity of the new method and to validate its ability in separating normal subjects from glaucomatous patients, the GGSS was then assessed in a different cohort of subjects, including 203 patients with OAG at various stages of severity (56 preperimetric [mean age 53.6 ± 14.1], 53 early [mean age 65.4 ± 10.9], 47 moderate [mean age 64.4 ± 8.3], and 47 advanced cases [mean age 67.8 ± 10.2], categorized according to the Hodapp–Parrish–Anderson method [3] to reduce any possible classification bias), and 43 normal subjects (mean age 57.9 ± 9.8). The MD mean values and the average RNFL thickness in the different groups are reported in Table 1.

The inclusion and exclusion criteria, testing methods, and OCT RNFL analysis protocol were identical to those used in the first part of the study. The eyes with a normal visual field and an abnormal optic disc, according to previously reported criteria, were considered as having preperimetric glaucoma. OCT data were not used in these cases to avoid any inclusion bias. Considering that glaucomatous optic disc damage can be difficult to detect in the early phases of the disease, any pathological change, based on optic disc photos and/or OCT images, during a follow-up period of at least 5 years was considered as a sign of a possible glaucomatous damage. Moreover, in most patients, the contralateral eye showed a definite damage due to glaucoma. The correlation between the GGSS and a six stage (plus a normal stage) clinical classification based on the visual field loss, staged with a modified Aulhorn and Karmeyer classification [10,11] and on the optic disc appearance, classified with the optic disc damage staging system [12], was assessed. The patients with a preperimetric glaucoma were analyzed separately. The receiver operating-characteristic (ROC) curves for MD, average RNFL thickness, GSS 2, OCT GSS, and the GGSS (considering as normal only the stage 0) in discriminating early glaucoma cases from healthy eyes were calculated.

The statistical analyses of the data were performed with the open-source software R (R Core Team, 2020). Statistical significance was defined as *p* < 0.05. 

## 3. Results

Considering the 147 cases with chronic glaucoma at different stages of severity (excluding the 56 preperimetric patients), the GGSS was able to correctly classify 89 cases (60.5%) within the same stage and 145 cases (98.6%) within the immediately adjacent stages (partial concordance), using the clinical classification as gold standard (Table 2).

Considering the 56 preperimetric cases, 54 (96.4%) were classified as borderline or abnormal in the Stage 1 or 2 by the GGSS.

**Figure 3:**
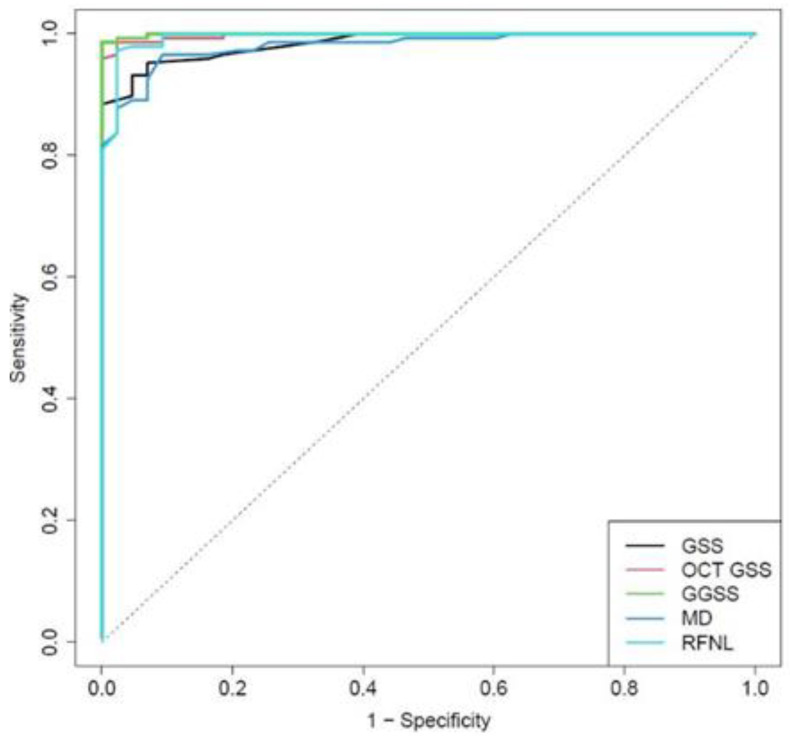
ROC curves for GGSS and the other methods used to classify all of the 147 glaucoma cases.

In the control group, 41 normal eyes out of 43 were correctly classified in the Stage 0, with a specificity of 95.3%. The GGSS ability to differentiate patients with glaucoma at any stage (excluding preperimetric eyes) from normal subjects was superior to MD, average RNFL thickness, GSS 2, and OCT GSS when taken in isolation. The area under receiver operating characteristic (ROC) curve for GGSS was 0.999, while for GSS 2, OCT GSS, MD, and RFNL, it was 0.984, 0.997, 0.981, and 0.994, respectively, with statistical significant differences for GGSS and MD (*p* = 0.016, *p* = 0.019) (Figure 3).

**Figure 4:**
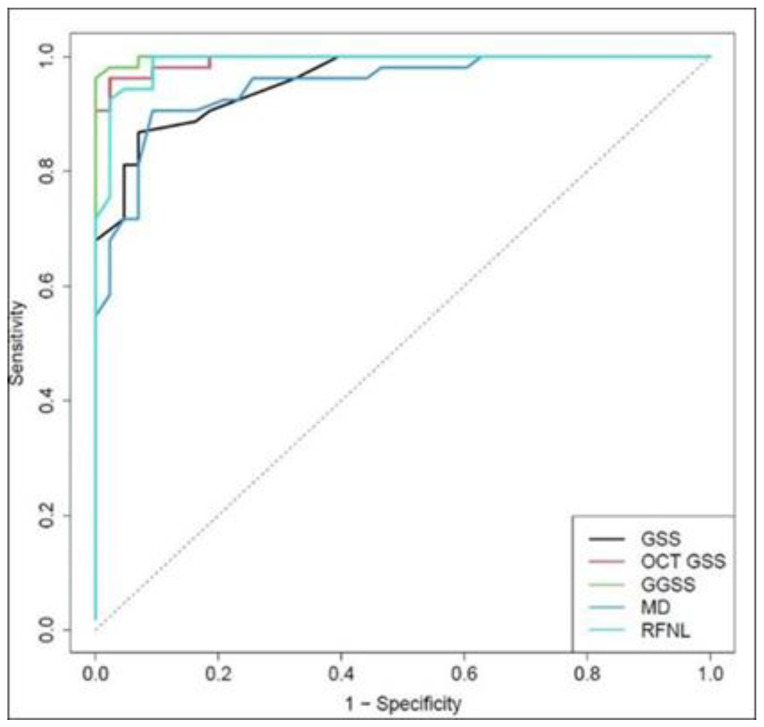
ROC curves for GGSS and the other methods used to classify the 53 early glaucoma patients.

From a clinical point of view, it should be highlighted that the GGSS ability to differentiate patients with early glaucoma (53 cases) from normal subjects was superior to MD, average RNFL thickness, GSS 2, and OCT GSS when considered in isolation. The area under ROC values were 0.998, 0.957, 0.993, 0.948, and 0.989 for GGSS, GSS 2, OCT GSS, MD, and RNFL, respectively (statistical significant differences for GGSS and MD; *p* = 0.013, *p* = 0.017) (Figure 4).

## 4. Discussion

The diagnosis of chronic glaucoma and the staging of damage are usually based on the information deriving from both the visual field testing, and the optic nerve and RNFL assessment currently performed with modern spectral domain OCTs. A clear relationship between perimetry results and structural loss can be found easily in moderate and advanced glaucoma, but this is not the case in the very early stages of the disease in which a significant structural loss can be detected in the presence of a normal visual field [13,14,15]. From a practical point of view, a lack of correlation between structure and function is not uncommon in clinical practice. Perhaps, as suggested by Medeiros and Tatham [16], it is now time to move beyond the old concept that contrasts structure and function, concentrating future studies on methods which are able to integrate different diagnostic information.

Special maps, showing the correlation between visual field test points and RNFL bundles, can help reduce measurement noise, which could have a practical role in glaucoma management [17,18,19,20,21].

Several structure-function indices, incorporating information from Heidelberg retina tomograph (HRT) or OCT and SAP, were introduced in the past few years [22,23,24,25,26,27]. Unfortunately, the software needed to obtain this information is not readily available for clinicians.

The ultimate goal in this field of research should be to create a system that is simple to use and able to integrate the information regarding the functional and structural damage.

Starting from the consideration that two reliable methods, the GSS 2 and the OCT GSS, are already available for an easy and standardized classification of functional and structural loss severity, the logical consequence was to attempt to integrate these two systems in order to obtain a global categorization of glaucomatous damage. The project presented many difficulties and obstacles, especially due to the need to reduce to the minimum the data provided by the two systems without losing the core of the information.

For this reason, this new method was designed to mainly concentrate mainly on the severity of damage, excluding information regarding the type of perimetric damage and the location of RNFL damage on OCT, which are in any case available on the final printout. Another obstacle was the fact that the two systems use rigid boundaries between stages; thus, results can significantly move and progress while still remaining in the same stage. To overcome this problem, the separation lines were removed, and only the actual values were taken into account in a continuous manner (i.e., a point in the middle of the Stage 3 was numbered as 3.5, etc.).

New software was created in which the user only needs to insert a few data. These include: name, date of birth, and eye of the patient; MD and PSD values; and RNFL superior and inferior quadrant thickness values.

**Figure 5:**
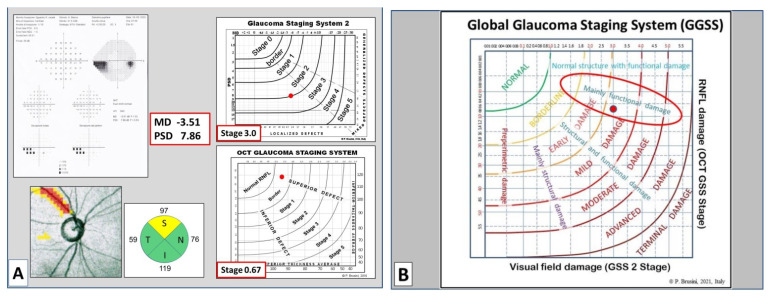
(**A**) 55 year old patient with primary OAG with a deep defect in the inferior nasal quadrant (GSS 2 Stage 3.0); OCT shows a subtle superior nerve fiber defect (OCT GSS Stage 0.67). This was considered as Stage 2, according to the clinical classification; (**B**) this case is classified as having a mild damage with a predominant functional defect.

**Figure 6:**
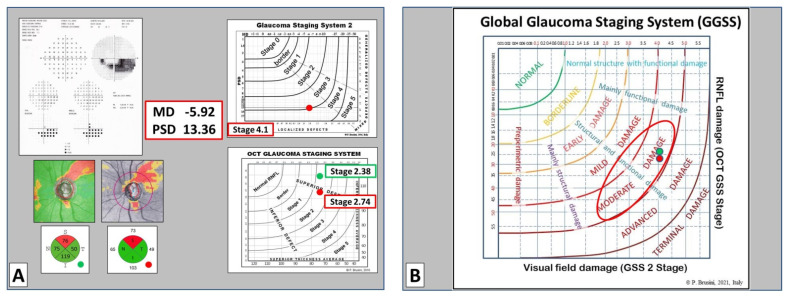
(**A**) 63 year old man with pseudoesfoliation glaucoma with a deep inferior arcuate scotoma (GSS 2 Stage 4.1); two different OCTs (Nidek RS-3000 on the left and Zeiss Cirrus on the right) show similar superior nerve fiber defect (OCT GSS Stage 2.38 and 2.74, respectively). Clinical classification: Stage 3; (**B**) the GGSS classifies this patient as having a moderate global damage with structural and functional loss.

**Figure 7:**
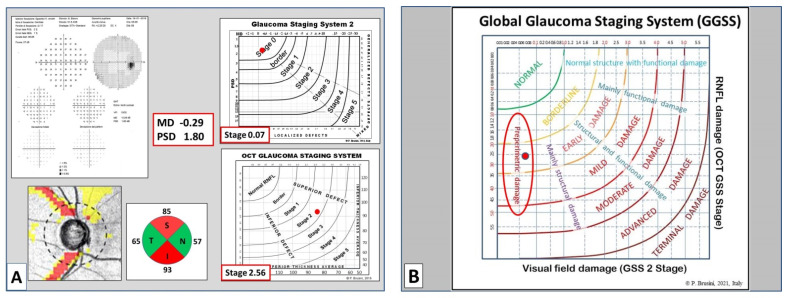
(**A**) 58 year old woman with primary OAG with a normal visual field (GSS 2 Stage 0.07) and a superior and inferior nerve fiber defect (OCT GSS Stage 2.56). This case was classified as Stage 1 using the clinical classification; (**B**) the GGSS classifies it as a preperimetric glaucoma at an early stage.

The GGSS immediately shows the severity of damage (in six stages), and its typology, in five classes of damage indicating whether the damage is predominantly functional or structural, or an equal combination of both (Figure 5, Figure 6 and Figure 7).

Considering that there is a continuum between these conditions, these sectors are not divided by lines, even if the software requires the use of regression lines to differentiate the different classes.

This software can process data from different automated perimeters (Zeiss–Humphrey, Octopus, Oculus, etc.), as long as the two main global indices (MD and PSD) are provided and can use data from almost all OCT machines currently on the market, as long as the RNFL thicknesses in the superior and inferior quadrant are available. The software automatically stages the visual field and RNFL damage (this information is separately provided in the printout) and, merging these data, indicates the global glaucomatous damage. This can be particularly useful in the early stages of the disease, when the visual field may be still normal (preperimetric glaucoma) or borderline. The ability of GGSS in differentiating normal subjects from patients having preperimetric or early glaucoma was assessed separately, considering that a correct clinical interpretation is often challenging in this type of patients. Even though glaucoma diagnosis and staging still rely on clinical assessment, the results of this study seem to prove that the GGSS may help less experienced ophthalmologists distinguish between healthy and diseased eyes, facilitating the formulation of a more reliable diagnosis and prognosis of the disease, decision making on the aggressiveness and type of treatment, and recording and storage of functional and structural data in a simple and clear format.

The opportunity of having homogeneous criteria for grading the severity of the disease can also be useful in research, in order to standardize the inclusion criteria in clinical studies in glaucoma, and for medical-legal purposes. The actual usefulness of this method in the follow-up of the progression of glaucomatous damage needs further investigation with prospective studies.

The GGSS has several limitations. First of all, the classification depends on only a few data, which can be affected by artifacts, subjectivity (for perimetry), inter- and intra-subject variability, image quality (for OCT), and other factors. Differences between various OCT machines could cause unequal classification of the same damage, but discrepancies are usually minimal and do not affect the clinical use of this method. Patients affected by normal-tension glaucoma were excluded, but this should not lead to significant variations in the results obtained. The subjects considered in this study were Caucasian; thus, these results should be used with caution in other ethnic populations. Normal subjects and patients with preperimetric glaucoma were younger compared to the other groups. This can be expected for patients having glaucoma in the early stages. With regards to the control group, it would have been preferable to take into consideration age-matched subjects, but this was difficult to achieve, due to the recruitment criteria used. This point can be considered a potential bias of this study. Moreover, this system does not take a possible macular ganglion cell loss without detectable RNFL thinning into consideration and thus can miss very early structural damage as demonstrated in recent studies [28,29], even if a recent systematic review of diagnostic accuracy studies [30] concludes that RNFL parameters are still preferable to macular parameters for diagnosing glaucoma. A modified version of GGSS is currently being studied, which considers the macular ganglion cell thickness when RNFL values are still within the normal limits. 

The lines which divide the various GGSS stages were determined using visual field tests and OCT data from hundreds of glaucomatous patients taking into account all the available clinical data and the final result appears to be satisfactory.

With regards to GSS 2 classification, even if is not ideal, it seems to work very well in comparison with other methods [31]. The OCT GSS has recently been introduced and clinical experience with it, using different OCTs, tends to be mostly positive. In this case, however, the lack of independent validation, with exception to a recent study [32], can be considered as a limitation. 

The new GGSS may give misleading information if the reliability of visual field testing or the quality of OCT images is not satisfactory. For this reason, the users should always look at the visual field reliability indices and imaging quality value (or signal strength index) before using the various classification methods. Unlike the GSS 2, but as per the OCT GSS, the GGSS requires specific downloadable software. It is possible, however, to manually enter data obtained with GSS 2 and OCT GSS onto a printable chart. In such cases of manual data entry, the user needs to estimate the partial stages to be recorded on the x and y axes.

Keeping these limitations in mind and remembering that this is a single-center study in which preliminary results need to be confirmed by other independent clinical studies, we believe the GGSS could become a useful graphic tool to integrate the way in which we assess and manage both functional and structural glaucomatous damage.

## 5. Patents

The Global Glaucoma Staging System software is patent pending (Patent Application No. 102019000006120). A link for downloading the GGSS software will be soon ready.

## Figures and Tables

**Table 1:** Mean MD value and average RNFL thickness in normal subject and in the four groups of patients considered for validation of the GGSS.

	Normal Subjects (*n* = 43)	Preperimetric Damage (*n* = 56)	Early Damage (*n* = 53)	Moderate Damage (*n* = 47)	Advanced Damage (*n* = 47)
**MD** (dB)	−0.9 ± 0.94	−0.84 ± 0.90	−2.3 ± 1.18	−6.4 ± 2.71	−16.0 ± 4.34
**RNFL** **Thickness** (microns)	107 ± 8.6	82.7 ± 8.3	75.9 ± 8.6	71.0 ± 9.4	56.9 ± 11.4

**Table 2:** Contingency table showing the agreement between the GGSS and the clinical classification. The numbers in bold indicate a perfect correspondence between the two classifications.

	GGSS						
**Clin Class**	0	0.5	1	2	3	3	5
0	**0**	0	0	0	0	0	0
0.5	0	**0**	0	0	0	0	0
1	1	4	**11**	12	1	0	0
2	0	0	9	**13**	5	0	0
3	0	0	0	6	**21**	8	0
4	0	0	0	0	4	**23**	3
5	0	0	0	0	0	5	**21**

Pearson Chi-Square *p* < 0.0001.

## Data Availability

The data presented in this study are available on request from the corresponding author.
